# Neurophysiological Correlates of Configural Face Processing in Schizotypy

**DOI:** 10.3389/fpsyt.2014.00101

**Published:** 2014-08-12

**Authors:** Rachel A. Batty, Andrew J. P. Francis, Hamish Innes-Brown, Nicole R. Joshua, Susan L. Rossell

**Affiliations:** ^1^Brain and Psychological Sciences Research Centre (BPsyC), Faculty of Health, Arts and Design, Swinburne University of Technology, Melbourne, VIC, Australia; ^2^School of Health Science, Psychology, RMIT University, Bundoora, VIC, Australia; ^3^The Bionics Institute, Melbourne, VIC, Australia; ^4^Pearson Clinical Assessment, Melbourne, VIC, Australia; ^5^Cognitive Neuropsychiatry Laboratory, Monash-Alfred Psychiatry Research Centre (MAPrc), The Alfred Hospital and Central Clinical School Monash University, Melbourne, VIC, Australia; ^6^Psychiatry, St. Vincents Hospital, Melbourne, VIC, Australia

**Keywords:** schizotypy, configural processing, face processing, N170, P100

## Abstract

**Background:** Face processing impairment in schizophrenia appears to be underpinned by poor configural (as opposed to feature-based) processing; however, few studies have sought to characterize this impairment electrophysiologically. Given the sensitivity of event-related potentials to antipsychotic medications, and the potential for neurophysiological abnormalities to serve as vulnerability markers for schizophrenia, a handful of studies have investigated early visual P100 and face-selective N170 in “at risk” populations. However, this is the first known neurophysiological investigation of configural face processing in a non-clinical schizotypal sample.

**Methods:** Using stimuli designed to engage configural processing in face perception (upright and inverted Mooney and photographic faces), P100 and N170 components were recorded in healthy individuals characterized by high (*N* = 14) and low (*N* = 14) schizotypal traits according to the Oxford–Liverpool Inventory of Feelings and Experiences.

**Results:** High schizotypes showed significantly reduced N170 amplitudes to inverted photographic faces. Typical N170 latency and amplitude inversion effects (delayed and enhanced N170 to inverted relative to upright photographic faces, and enhanced amplitude to upright versus inverted Mooney faces), were demonstrated by low, but not high, schizotypes. No group differences were shown for P100 analyses.

**Conclusions:** The findings suggest that neurophysiological deficits in processing facial configurations (N170) are apparent in schizotypy, while the early sensory processing (P100) of faces appears intact. This work adds to the mounting evidence for analogous neural processing anomalies at the healthy end of the psychosis continuum.

## Introduction

Given the social cognitive anomalies characteristic of schizophrenia, emotion processing has received substantial research attention (1–6). Overwhelmingly, significant impairments in emotion perception are reported (7), and these appear present and stable from pre-onset to chronic multi-episode patients (2). A more recent line of inquiry has suggested that deficient facial emotion processing in schizophrenia may be underpinned by basic visuoperceptual deficits (8, 9), although electrophysiological evidence of this is not always demonstrated. Studies using neutral face stimuli have verified a primary deficit in the processing of configural information (described below), with a relative overreliance on facial feature processing by patients with schizophrenia (10, 11), and in ultra-high risk individuals (12). This appears to extend to non-face processing as well (11, 13), supporting a generalized bias for local relative to global perceptual processing.

In the context of face perception, configural processing refers to (i) the basic detection of the face formation (i.e., eyes above nose above mouth; first-order relations), (ii) the uniting of these as a gestalt or whole image (holistic processing), and (iii) an assessment of the spatial relationships between facial features, thought to underlie identity processing (second-order relations) [see Ref. (14, 15)]. The disruption of configural processing when a face is inverted produces the “face-inversion effect” (FIE) (16): upside-down faces are more difficult to perceive, discriminate, and recognize, demonstrated by a decrease in accuracy and increase in reaction times (RT) compared with upright faces, first reported by Yin (17). The FIE has been researched extensively [e.g., Ref. (14, 16, 18, 19)], and in schizophrenia the effect is often absent, aligned with evidence for a configural processing deficit (20–22), however, see Ref. (23) for evidence of the FIE in patients).

The different stages of face processing are reflected by the P100 and N170 event-related potentials (ERPs). The P100 component is an occipitally distributed positive deflection, with a typical peak latency between 80 and 120 ms, and is associated with early stages of visual information processing (24, 25). The N170 component is maximal over the ventral occipitotemporal cortex with a peak latency between 140 and 200 ms post stimulus onset. N170 amplitude is consistently larger for faces compared to other objects, and for this reason has been considered “face-selective” (26–28). Various attempts have been made to define N170 face-specificity further, for instance, in response to eyes only (26, 29, 30), facial emotion (31–34), and identity encoding (28). One group has even argued controversially against N170 face selectivity (35), however, most evidence points toward an index of face-specific early cortical processing (36–41).

The N170 is also modulated by configural face processing, with effects reported in response to whole faces, but not half faces (42), schematic faces that provide spatial face configuration but no distinguishable featural face information (i.e., first-order configural information) (14, 43) and two-tone Mooney faces (44) that rely on holistic processing (global gestalt) to be perceived (14, 45, 46). Reliable modulation of the N170 component is also demonstrated by the FIE: upside-down faces consistently elicit a delayed latency and enhanced amplitude over usual N170 occipitotemporal electrodes, relative to upright faces (14, 26, 43, 47). This is generally regarded as further evidence of N170 sensitivity to configural face information. Although the N170 effects in response to the inversion of schematic and Mooney faces are less consistent, a delayed and reduced N170 to upside-down schematic faces (14, 48), and reduced N170 amplitude to upside-down Mooney faces (45, 46) have been shown.

Reductions in P100 amplitude to various visual stimuli have been demonstrated in patients with schizophrenia (49–51) as well as in unaffected first-degree relatives (52), those with an “at risk” mental state (53), in schizotypy (54), and in non-pathological healthy individuals prone to visual hallucinations (55). This suggests an association between schizophrenia and impoverished visual input, and is supported by existing patient deficits in attention (56–58), as well as visual scan paths characterized by fewer visual fixations, longer duration of fixations/saccades, and smaller saccade amplitudes (21, 59, 60). However, P100 deficits have not always been reported in patient studies (13, 61–64), or in schizotypy (25). It is also noteworthy that P100 effects have typically been recorded in response to basic visual stimuli (i.e., isolated gray/white check images and line drawings) (50–52, 65, 66), with only a handful of studies demonstrating P100 deficits to (emotional) face stimuli in patients (49, 67), and in those at risk for psychosis (53). Last, antipsychotic agents have known effects on neural activation (68, 69). An increase in P100 latency during visual discrimination has previously been shown following an acute dose of bromazepam (70).

In contrast, N170 studies in schizophrenia, although few, have consistently demonstrated reduced N170 amplitude (34, 49, 63, 67, 71), and delayed N170 latency (49) relative to healthy samples. However, N170 amplitude reductions have only been shown in an at-risk population by one study (53), with no evidence for N170 effects reported in first-degree relatives (34), and in individuals prone to visual hallucinations (55). This is surprising given the hereditary nature and spectrum account of psychosis. Shared neurocognitive deficits are commonplace in healthy yet prone individuals (72–76), and the potential for neural markers to serve as endophenotypes in schizophrenia has been established [e.g., Ref. (77, 78)]. Thus, further evidence is necessary to determine whether face processing deficits illustrated neurophysiologically at N170 in patients are shared by individuals prone to psychosis.

Moreover, with rare exception [i.e., Ref. (55, 79)], the N170 literature has notably used emotional face stimuli (34, 49, 53, 63, 67, 71, 80). Thus, more evidence for the ERP correlates of configural face processing, without the potentially confounding positive and negative valence information, is also necessary. Given the established effect of pharmacological agents on neural activation (68–70), individuals prone to psychosis, and medication naïve, provide an ideal method of investigating analogous neural processing deficits (73, 74, 81–85), without concern for medication, and other potential confounds introduced by clinical samples (i.e., long-term hospitalization, social isolation) (82, 86). With this in mind, schizotypy provides a valuable model of investigation. To our knowledge, the N170 response in schizotypy has not yet been reported.

This study aimed to expand on existing literature by avoiding emotionally laden stimuli and clinical confounds while recording neural markers of face processing. Using stimuli designed to engage configural processing in face perception (upright and inverted Mooney and photographic faces), we sought to determine the ERP correlates (P100, N170 components) of configural face processing in schizotypy. We expected that, in high schizotypes, reduced P100 amplitudes would indicate impoverished visuosensory input, whereas reduced N170 amplitudes would indicate impaired face processing. Anomalous ERP responses to (i) Mooney faces, and (ii) inverted stimuli of both types, would provide evidence of configurally specific face processing deficits.

## Materials and Methods

### Participants

Thirty participants (15 male), between ages 18 and 55 years were recruited from RMIT University, Melbourne and the Mental Health Research Institute (MHRI) participant database. Two (1 male) were excluded from the N170 analyses due to (i) inadequate accepted trials (inverted Mooney stimuli), and (ii) a corrupted data file (*M* = 27.24 years, SD = 7.48, 14 male). A third was removed from the P100 analyses due to poor quality recording on principal electrode OZ (*M* = 27.20 years, SD = 7.62, 14 male). All had normal or corrected to normal visual acuity, IQ within the average range [National Adult Reading Test IQ; NART; (87)], no concurrent alcohol or substance abuse, and no personal or family history of psychopathology (self-report).

#### Schizotypal personality

The Oxford–Liverpool Inventory of Feelings and Experiences [O-LIFE; (88)] was completed as a measure of psychosis-proneness for each participant. The O-LIFE is a 159 yes/no item self-report questionnaire, which measures four distinct schizotypy dimensions with high internal consistency: unusual experiences (α = 0.89), cognitive disorganization (α = 0.87), introvertive anhedonia (α = 0.82), and impulsive non-conformity (α = 0.77) (89). A median split of the O-LIFE Cognitive Disorganization dimension defined high/low schizotypy groups. Cognitive Disorganization includes deficits in attention, concentration, decision making, and social anxiety, and the scale was deemed most appropriate because it assesses traits that reflect these cognitive deficits as well as the positive symptoms of psychosis (88, 90)^1^. Moreover, self-face recognition failures correlate with cognitive perceptual/disorganized schizotypy dimensions (90). Groups were matched on NART IQ (see Table 1).

**Table 1:** **Demographic characteristics of high and low schizotypy**.

	Mean (Standard Deviation)			
	P100		N170	
	Low schizotypy (*n* = 13)	High schizotypy (*n* = 14)	Low schizotypy (*n* = 14)	High schizotypy (*n* = 14)
Age	30.16 (9.69)	24.45 (3.54)*	30.03 (9.32)	24.46 (3.54)*
Gender (M/F)	6∕7	8∕6	6∕8	8∕6
NART IQ	108.15 (8.65)	104.29 (8.11)^#^	108.43 (8.37)	104.29 (8.11)^†^
O-LIFE scales
Unusual experiences	4.46 (5.36)	7.64 (5.89)	4.14 (5.29)	7.64 (5.89)
**Cognitive disorganization**	**4.46 (2.30)**	12.57 (4.27)***	**4.21 (2.39)**	12.57 (4.27)***
Introvertive anhedonia	2.00 (1.29)	5.43 (3.03)***	1.93 (1.27)	5.43 (3.03)***
Impulsive non-conformity	6.31 (2.94)	9.36 (3.30)*	6.14 (2.88)	9.36 (3.30)**

*O-LIFE; Oxford–Liverpool Inventory of Feelings and Experiences (88). High and low schizotypy was defined by the Cognitive Disorganization dimension (M[SD] values in bold font)*.

*The significant age difference reflects an outlier (*n* = 1) in the low schizotypy group (results did not change when this outlier was removed)*.

***p < 0.001; **p < 0.01; *p < 0.05.

*^#^*p* = 0.24 ^†^*p* = 0.20*.

### Face recognition tasks

Two computerized tasks (20 min duration) were completed during electroencephalographic (EEG) recording. These were counterbalanced, and stimulus order was randomized for each participant. A short break was given after each 10 min block. Task One stimuli were a series of 40 original Mooney faces (44). These were digitally manipulated and repeated to create four separate conditions: *upright face, inverted face, upright disorganized face*, and *inverted disorganized face*^2^. A total of 640 stimuli were presented, with 160 per condition. Twelve^3^ neutral grayscale photographic faces were used as Task Two stimuli [Ekman and Friesen series, (91)]. The same four conditions as in Task One were created, with a total of 576 stimuli presented (144 per condition).

All participants were shown a printed example of each condition type prior to the task. They fixated on central fixation cross with a random duration from between 800 and 1200 ms between stimuli, and were shown the images for 200 ms (stimuli were thus on screen for the duration of the critical time period for both P100 and N170). Using a two-button control, participants indicated when they saw either an intact (left button) or disorganized (right button) face. Accuracy and RTs were recorded. These data were submitted to repeated measures analysis of variance (ANOVA) with task (Mooney and photographic faces) and orientation (upright and inverted) as within-subjects factors, and schizotypy (high and low) as the between subjects factor.

### Electrophysiological acquisition and data processing

Electroencephalographic activity was recorded continuously from 64 scalp sites (10/20 International system, *Neuroscan 4.2*, amplified using *SynAmps2* system). Recording sites included eight midline electrodes (FPZ, FZ, FCZ, CZ, CPZ, PZ, POZ, OZ), 28 electrodes over each hemisphere (FP1/FP2, AF3/AF4, AF7/AF8, F1/F2, F3/F4, F5/F6, F7/F8, FC1/FC2, FC3/FC4, FC5/FC6, FT7/FT8, C1/C2, C3/C4, C5/C6, T7/T8, CP1/CP2, CP3/CP4, CP5/CP6, TP7/TP8, P1/P2, P3/P4, P5/P6, P7/P8, PO3/PO4, PO5/PO6, PO7/PO8, O1/O2, CB1/CB2), and the left and right mastoids. A nose reference was used during acquisition and an average reference montage was calculated offline. The midline electrode between FPZ and FZ served as the ground. Electrooculogram (EOG) was measured at FP1.

Signals were amplified 20,000× and digitized at a sampling rate of 1000 Hz with a band-pass filter of 0.1–100 Hz (24 dB/octave; zero phase shift). Digital codes were sent from the stimulus-presentation computer, and response button-press, to mark the onset and type of each stimulus, and the participant response, respectively. Movement-contaminated EEG sections were discarded, and continuous data files were corrected for eye-blinks and divided into epochs from 100 ms pre-stimulus to 500 ms post-stimulus. Following baseline correction, epochs with artifacts that exceeded ±100 μV were rejected. Only trials with the correct behavioral responses (*N* > 20 p/condition)^4^ were included and filtered at 0.5–35 Hz (24 dB/octave; zero phase shift) (Table 2). ERPs were created by averaging together stimuli of the same condition subtype.

**Table 2:** **Mean (SD) accepted trials per condition**.

	Mooney		Photographic	
	Upright	Inverted	Upright	Inverted
P100 analyses
Low schizotypy	122.85 (60.40)	73.08 (44.78)	109.46 (59.26)	109.38 (53.64)
High schizotypy	131.93 (65.81)	80.43 (48.95)	137.86 (70.97)	106.07 (28.58)
N170 analyses
Low schizotypy	123.21 (58.04)	75.07 (43.70)	110.64 (57.11)	106.57 (52.60)
High schizotypy	131.93 (65.81)	80.43 (48.95)	137.86 (70.97)	106.07 (28.58)

*Reduced accepted trials for P100 analyses reflects the removal of a dataset (*n* = 1) due to poor quality recording on principal electrode OZ*.

### Data analysis

Component P100 was measured as the maximal positive deflection between 80 and 120 ms (25) at electrodes O1, OZ, and O2 [established optimal occipital scalp sites; (25, 51, 53, 67, 70)]. Peak latencies and amplitudes from baseline were submitted to repeated measures ANOVA, with task (Mooney and photographic faces) and orientation (upright and inverted) as within-subjects factors. The N170 was measured as the maximal negative deflection between 140 and 200 ms (14, 28, 45) at PO7 and PO8 (41, 48). Peak N170 latencies and amplitudes from baseline were submitted to repeated measures ANOVA, with task (Mooney and photographic faces), orientation (upright and inverted), and hemisphere (left and right) as within-subjects factors. High and low schizotypy served as the between subject factor for all analyses. The Greenhouse–Geisser epsilon correction factor was applied to account for possible effects of non-sphericity where appropriate. To further investigate amplitude differences at N170, independent sample *t-*tests were run using the mean amplitude across PO7/PO8 components. Relationships between ERP data and O-LIFE scores were investigated by Spearman’s correlation coefficients.

## Results

### Behavioral data

An adequate number of trials remained for all but one participant. Two others had accepted trials in the 20s, and the remainder had >37 (Table 2). The accuracy and RT data are presented in Table 3. Participants correctly identified a greater number of photographic than Mooney faces; *F*(1,26) = 146.77, *p* < 0.001, $\eta_{p}^{2}=0.85$ and a greater number of upright than inverted faces; *F*(1,26) = 147.77, *p* < 0.001, $\eta_{p}^{2}=0.85$. A task × orientation interaction reflected a large decline in accuracy for the inverted Mooney faces, not shown to the inverted photographic faces; *F*(1,26) = 124.47, *p* < 0.001, $\eta_{p}^{2}=0.85$. These findings were mirrored by RTs: participants responded faster to photographic than Mooney faces; *F*(1,26) = 62.68, *p* < 0.001, $\eta_{p}^{2}=0.85$ and faster to upright than inverted faces; *F*(1,26) = 197.99, *p* < 0.001, $\eta_{p}^{2}=0.85$. A task × orientation interaction once again reflected much slower responses to inverted Mooney faces; *F*(1,26) = 24.94, *p* < 0.001, $\eta_{p}^{2}=0.85$. Neither accuracy nor RT differentiated the schizotypy groups: accuracy, task *p* = 0.33, orientation *p* = 0.64; and RTs, task *p* = 0.76, orientation *p* = 0.58 (Table 3).

**Table 3:** **Mean (SD) accuracy and reaction times per condition**.

	Mooney		Photographic	
	Upright	Inverted	Upright	Inverted
% correct
Low schizotypy	87.2(4.7)	53.9 (17.7)	96.9 (2.8)	94.7 (6.0)
High schizotypy	83.8 (12.6)	48.3 (18.9)	97.2(2.6)	94.4 (4.7)
RTs (ms)
Low schizotypy	696.1 (99.7)	809.8 (128.9)	638.2 (91.4)	687.2 (106.9)
High schizotypy	690.4 (56.5)	791.1 (97.0)	632.5 (58.7)	681.9 (49.7)

### Event-related potentials

#### P100

**Figure 1:**
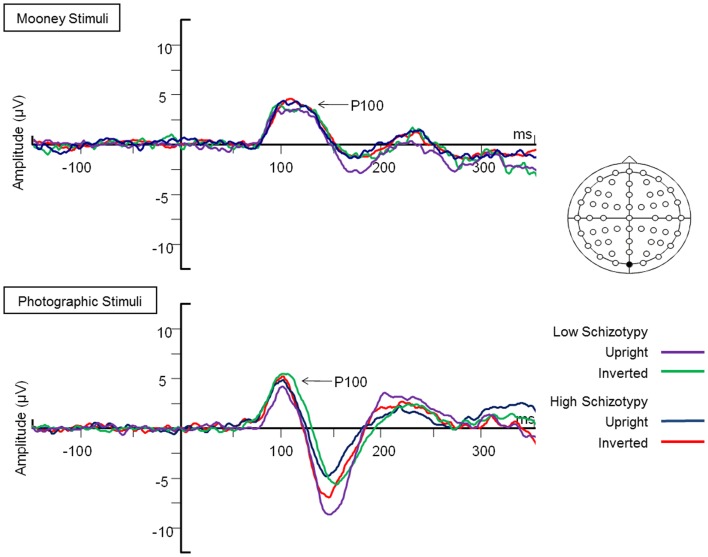
**Grand-averaged P100 waveforms at electrode OZ**. Upright and inverted stimuli presentations across groups are compared for both tasks. A bird’s eye view of the electrode montage is shown with the plotted electrode shaded black. Negative polarity is plotted downward.

Mean (SD) amplitudes and latencies are presented in Table 4, and grand-averaged waveforms are illustrated in Figure 1. P100 latency was increased for inverted relative to upright faces at electrode O1; *F*(1,25) = 6.22, *p* = 0.02, $\eta_{p}^{2}=0.85$. Larger amplitudes were shown to photographic than Mooney faces at all three occipital sites: (i) O1; *F*(1,25) = 10.20, *p* = 0.004, $\eta_{p}^{2}=0.85$ (ii) OZ; *F*(1,25) = 10.81, *p* = 0.003, $\eta_{p}^{2}=0.85$ (iii) O2; *F*(1,25) = 8.68, *p* = 0.007, $\eta_{p}^{2}=0.85$. Greater amplitude to inverted versus upright faces was shown at electrode O2 only; *F*(1,25) = 14.74, *p* = 0.001, $\eta_{p}^{2}=0.85$ (trend level at OZ, *p* = 0.06). No differences between schizotypal groups were shown for P100 latency: (i) O1; *p* = 0.85, (ii) OZ; *p* = 0.54, (iii) O2; *p* = 0.61, or P100 amplitude: (i) O1; *p* = 0.19, (ii) OZ; *p* = 0.63, (iii) O2; *p* = 0.35. No other significant P100 effects were shown.

**Table 4:** **P100 Mean (SD) amplitude and latency per condition and electrode**.

	Mooney						Photographic					
	Upright			Inverted			Upright			Inverted		
	O1	OZ	O2	O1	OZ	O2	O1	OZ	O2	O1	OZ	O2
Latency (ms)
Low schizotypy	103.08 (11.28)	98.62 (6.61)	102.38 (11.36)	106.38 (12.43)	97.23 (8.08)	101.00 (12.39)	102.08 (9.10)	100.23 (9.86)	101.85 (8.74)	105.92 (7.57)	97.38 (9.28)	102.92 (7.94)
High schizotypy	107.21 (9.70)	94.29 (7.15)	104.93 (11.50)	108.79 (9.56)	97.00 (8.27)	106.43 (10.77)	100.50 (8.22)	101.07 (8.90)	101.43 (7.60)	102.93 (9.44)	95.50 (8.21)	101.21 (6.34)
Amplitude (μV)
Low schizotypy	6.75 (3.72)	2.59 (2.70)	5.31 (2.19)	6.84 (2.95)	2.46 (2.45)	6.50 (3.33)	8.62 (3.56)	3.75 (3.19)	7.91 (3.04)	8.56 (4.23)	4.90 (3.72)	8.31 (2.99)
High schizotypy	5.66 (2.79)	2.61 (2.53)	5.24 (2.41)	5.84 (2.79)	2.61 (2.31)	6.36 (3.48)	6.26 (3.21)	2.80 (2.64)	6.03 (2.72)	6.67 (3.34)	3.90 (2.42)	6.73 (3.16)

*Low schizotypy (*n* = 13), high schizotypy (*n* = 14)*.

#### N170

##### Latency

**Figure 2:**
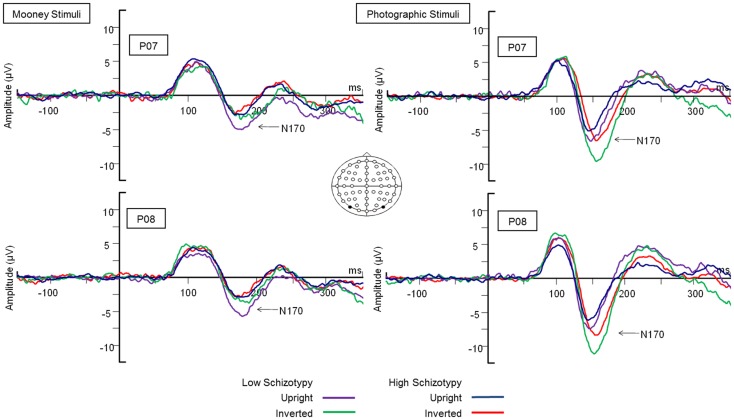
**Grand-averaged N170 waveforms at electrodes PO7 and PO8**. Upright and inverted stimuli presentations across groups are compared for both tasks. A bird’s eye view of the electrode montage is shown with the plotted electrodes shaded black. Negative polarity is plotted downward.

**Figure 3:**
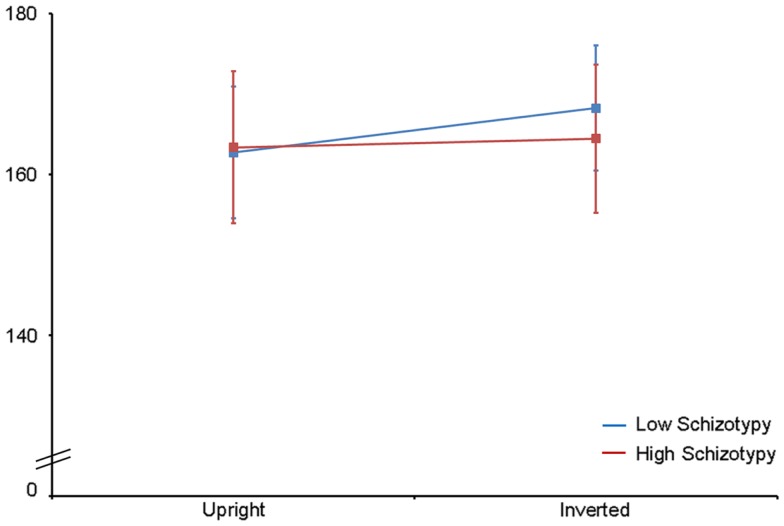
**N170 latency group × orientation interaction effect**. Comparable peak latencies are shown to upright stimuli by both groups, however, high schizotypes show earlier peak latencies to inverted face stimuli (most difficult to perceive).

Mean (SD) amplitude and latency to upright and inverted stimuli for both tasks are shown in Table 5, and N170 waveforms at P07/08 are shown in Figure 2. Earlier N170 latencies were shown to photographic (*M* = 154.72 ms, SD = 10.06), than to Mooney (*M* = 174.63 ms, SD = 10.33) faces: *F*(1,26) = 79.52, *p* < 0.001, $\eta_{p}^{2}=0.85$ and to upright (*M* = 163.02 ms, SD = 8.68) than inverted (*M* = 166.33 ms, SD = 8.61) faces: *F*(1,26) = 18.67, *p* < 0.001, $\eta_{p}^{2}=0.85$. A task × orientation interaction demonstrated similar latencies to upright and inverted Mooney faces, whereas upright photographic faces were marked by earlier latencies relative to inverted photographic faces: *F*(1,26) = 8.83, *p* = 0.006, $\eta_{p}^{2}=0.85$ (see Table 5). The left hemisphere showed earlier latencies (*M* = 162.54 ms, SD = 9.54), than the right (*M* = 163.50 ms, SD = 9.09) to upright faces, whereas this effect was reversed for inverted faces where earlier latencies were shown in the right hemisphere (*M* = 165.20 ms, SD = 9.38) versus left (*M* = 167.46 ms, SD = 9.36): orientation × hemisphere interaction, *F*(1,26) = 4.69, *p* = 0.04, $\eta_{p}^{2}=0.85$. While there was no main effect for schizotypy group (*p* = 0.63), a group × orientation interaction was shown. The low schizotypy group had earlier latencies for upright relative to inverted faces; however, the high schizotypy group had comparable latencies across orientations: *F*(1,26) = 8.41, *p* = 0.007, $\eta_{p}^{2}=0.85$ (see Table 5; Figure 3). A task × orientation × group interaction was at trend level (*p* = 0.067).

**Table 5:** **N170 Mean (SD) amplitude and latency per condition and electrode**.

	Mooney				Photographic			
	Upright		Inverted		Upright		Inverted	
	PO7	PO8	PO7	PO8	PO7	PO8	PO7	PO8
Latency (ms)
Low schizotypy	173.57 (12.07)	174.64 (9.83)	179.86 (11.27)	177.14 (13.33)	151.79 (7.91)	150.79 (10.37)	158.64 (9.13)	157.29 (11.50)
High schizotypy	173.79 (11.90)	176.29 (14.13)	173.29 (13.50)	168.43 (10.79)	151.00 (15.37)	152.29 (15.33)	158.07 (11.68)	157.93 (9.96)
Amplitude (μV)
Low schizotypy	− 6.13 (3.81)	−6.78 (3.61)	− 5.25 (4.13)	−5.25 (4.46)	− 8.10 (4.91)	−8.79 (3.77)	− 11.21 (3.98)	−12.89 (4.12)
High schizotypy	− 3.98 (3.34)	−4.08 (3.69)	− 3.96 (2.36)	−4.07 (2.85)	− 6.55 (4.46)	−7.57 (6.19)	− 8.02 (4.56)	−9.55 (6.01)

##### Amplitude

**Figure 4:**
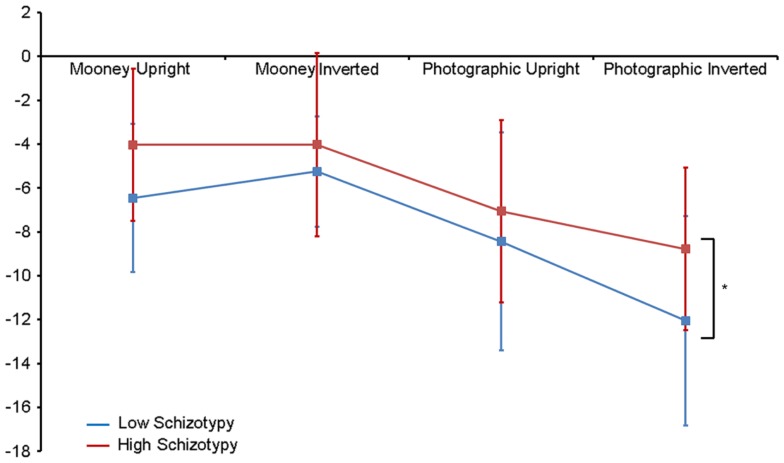
**N170 amplitude group × task × orientation interaction effect**. Both high and low schizotypes showed the typical inversion effect to photographic stimuli: larger N170 amplitude to inverted versus upright photographic faces (although this difference is smaller for the high schizotypes). However, only low schizotypes showed an inversion effect to Mooney faces (reversed: smaller amplitudes to inverted compared with upright Mooney faces). High schizotypes instead showed comparable amplitude to Mooney faces irrespective of orientation. Note the overall trend for reduced amplitudes in high schizotypy. *p = 0.05.

Greater N170 amplitude was shown to photographic (*M* = − 9.08 μV, SD = 4.34) than to Mooney (*M* = − 4.94 μV, SD = 3.36) faces: *F*(1,26) = 46.18, *p* < 0.001, $\eta_{p}^{2}=0.85$ and to inverted (*M* = − 7.53 μV, SD = 3.48) relative to upright (*M* = − 6.50 μV, SD = 3.71) faces: *F*(1,26) = 18.23, *p* < 0.001, $\eta_{p}^{2}=0.85$. A task × orientation interaction also demonstrated that N170 amplitudes were greater for upright Mooney faces (relative to inverted), however, amplitudes were greater for inverted photographic faces (relative to upright): *F*(1,26) = 22.15, *p* < 0.001, $\eta_{p}^{2}=0.85$ (see Table 5). Furthermore, amplitudes were comparable across hemisphere for Mooney faces in both orientations, but greater in the right hemisphere for photographic faces, especially in the inverted orientation: task × orientation × hemisphere interaction, *F*(1,26) = 4.70, *p* = 0.04, $\eta_{p}^{2}=0.85$ (see Table 5). Again, while there was no main effect for schizotypal group (*p* = 0.12), a group × task × orientation interaction was shown: *F*(1,26) = 4.87, *p* = 0.04, $\eta_{p}^{2}=0.85$. The low schizotypy group demonstrated increased amplitude to upright versus inverted Mooney faces and substantially increased amplitude to inverted versus upright photographs. However, the high schizotypy group demonstrated comparable amplitude to Moony faces in both orientations, and only marginally increased amplitude to inverted versus upright photographs (Table 5; Figure 4). *Post hoc* analyses using independent sample *t*-tests were run on the accumulated N170 mean amplitude [i.e., (PO7 + PO8)/2] for Mooney upright, Mooney inverted, photographic upright, and photographic inverted, separately. The high schizotypy group showed significantly reduced N170 amplitudes for inverted photographic faces only; *t*(26) − 2.02, *p* = 0.05, *d* = 0.77 (Mooney upright, *p* = 0.07, Mooney inverted, *p* = 0.35, and photographic upright, *p* = 0.43).

### ERP correlations with O-LIFE scores

Oxford–Liverpool Inventory of Feelings and Experiences scores from the entire sample (*n* = 28) were negatively correlated with the latency of the N170 for inverted Mooney faces, where higher scores (i.e., greater schizotypy) was associated with earlier peak latency for the inverted Mooney faces (*r* = − 0.38, *p* = 0.05). No other correlations were significant.

## Discussion

N170 latency and amplitude main effects reflected the established literature (14, 45, 46), that is, earlier N170 latencies were demonstrated to photographic (relative to Mooney) and upright (relative to inverted) faces. This typically indicates the more efficient information processing of stimulus categories that are easier to perceive (i.e., photographic and upright faces). N170 amplitude was also larger to both photographic (relative to Mooney) and inverted (relative to upright) faces. The interaction effect clarified that peak amplitudes were greater for upright compared with upside-down Mooney faces, whereas the opposite was true for photographs: these showed the classic inversion effect of larger amplitude to upside-down compared with upright photographic faces. These amplitude effects are discussed in detail with respect to the schizotypal group differences [for more information, see (14, 45)].

Our data demonstrate that individuals high in schizotypal traits show significantly reduced N170 amplitudes to inverted photographic faces. This finding is consistent with the limited N170 literature in schizophrenia (34, 49, 63, 67, 71), and in at-risk individuals (53) where emotional face stimuli has been used. A similar, though non-significant, pattern of reduced N170 amplitude was demonstrated for the remaining three face categories (upright photographic, and upright and inverted Mooney). It is unclear why these categories did not reach significance. In a study using comparable stimuli to ours, Schwartzman et al. (55) also reported no N170 amplitude differences between individuals with high and low proneness to visual hallucinations. However, as the authors suggest, this is probably because deficits in hallucination-prone individuals are more likely to be visuo-sensory specific (as was reflected by P100 differences in their sample), and less likely to be face-specific. In schizotypy, however, neurocognitive deficits in attention, perception, social anxiety, and cognitive disorganization are shared with patients, making them more liable to face-specific deficits (88, 90). Neural processing anomalies shared by healthy individuals prone to psychosis, which are likely to be reduced in degree, may only be detected where effects are especially robust. In our study, this was demonstrated to inverted photographic faces, which are renowned for eliciting a strong amplitude response (14, 26, 43, 47).

Individuals low in schizotypal traits demonstrated the classic increase in amplitude to inverted relative to upright photographs, and an increase in amplitude to *upright* relative to inverted Mooney faces. This latter amplitude effect to Mooney faces has been shown previously, especially on trials where stimuli are recognized as a face (45, 46), which was also the case here. It has been proposed that upright photographic faces engage all three stages of configural processing; first-, holistic, and second-order (14). However, when upside-down, configural processing is disrupted and these faces are processed analytically (i.e., a part by part process using their featural information), which explains the reliably demonstrated increase in N170 amplitude in response to inversion (45). Similarly, Mooney faces containing configural (holistic/gestalt) information are only processed holistically when presented upright, accounting for a smaller N170 amplitude when compared to upright photographs. Upon inversion, however, featural information is unavailable in the Mooney face, and so analytic processing is not engaged. The subsequent difficulty of processing Mooney faces holistically when upside-down is demonstrated by the reduction in N170 amplitude (45). These typical N170 effects were expected from individuals low in schizotypal traits, and are further reflected by their earlier N170 peak latencies to upright compared with inverted faces, indicating faster face processing to upright faces.

By contrast, the high schizotypes in our study demonstrated comparable N170 amplitude to Mooney faces in both orientations, only marginally increased amplitude to inverted versus upright photographs, and comparable peak latencies across orientations to both face types. Face processing for the high schizotypes was thereby significantly less affected by orientation. Thus, this group was less affected by the disruption to configural information processing in inversion, supporting the established generalized bias for local as opposed to global perceptual processing in schizophrenia (10, 11, 13), and in psychosis prone individuals (12). This is further suggested by the relationship shown between N170 latency and O-LIFE scores for inverted Mooney faces, which indicated that the speed of information processing (latency) increased as schizotypal traits increased. Inverted Mooney faces are the most difficult stimulus category to perceive because configural information is disrupted but alternate featural processing cannot be engaged. The fact that schizotypal traits are associated with faster processing of these stimuli demonstrate further that face processing in high schizotypes is less reliant on configural processing. The generalized poor recruitment of configural information processes may further explain the overall trend for reduced N170 amplitude in this group. However, their neural response to the photographic stimuli suggests that while high schizotypes may have a bias for featural/local processing, they may not be expert in this method of processing either. If this were the case, expertise in part by part analytic processing should be shown electrophysiologically in this group in response to photographic faces. According to the existing literature, a typical, but enhanced, spike in N170 peak amplitude would be expected, and would likely exceed that of the low schizotypes in both orientations. Instead, high schizotypes showed the opposite of this: generally (though non-significantly) reduced amplitude to upright photographs and significantly reduced amplitude in response to photographic faces presented upside-down.

The P100 component is sensitive to changes in luminance and contrast (96). Thus, larger P100 amplitudes to photographic faces in our study reflects added visuosensory input compared with that of the basic black and white shaded Mooney face. Latinus and Taylor (14) have previously reported no differences in P100 latency or amplitude between photographic and Mooney face stimuli, although, they did observe an amplitude decrease to schematic faces, which supports this interpretation. In our study, the demonstrated sensitivity of P100 to orientation (i.e., reduced amplitude and increased latency for inverted versus upright stimuli) is less intuitive. Stimulus characteristics remain consistent across orientations, with more advanced stimulus discrimination not generally shown until later time windows [e.g., N170, N250; see Ref. (37)]. However, P100 may also be modulated by the allocation of attentional resources (70, 96), and it would stand to reason that attention may decline for upside-down faces over the duration of the task, which could explain this finding. Importantly, the absence of schizotypal group differences at P100, as well as the lack of relationship between P100 and O-LIFE scores, demonstrates that early visuosensory processing in high schizotypy appears intact.

Despite the aforementioned significant neurophysiological anomalies, our behavioral data reinforced the healthy status of these individuals high in schizotypal traits. Behavioral responses conformed to previous findings for both high and low schizotypes: stimuli easier to recognize (i.e., photographic and upright faces) attracted more accurate and faster responses, with the least accurate and slowest responses demonstrated for faces most difficult to perceive (i.e., inverted Mooney faces) (14, 45, 46, 55). It is not unusual that high and low schizotypes show matched behavioral performance. Semantic priming literature in schizotypy has consistently demonstrated ERP differences in high and low schizotypes that are not reflected behaviorally [e.g., Ref. (92–94)]. It has been argued that this is because behavioral measures capture later stages of processing, by which time anomalies in neural processing have been accounted for in healthy brains [see Ref. (95) for discussion].

In summary, high schizotypes demonstrated impaired face processing (N170 component), which appears to stem from a specific deficit in the configural assessment of faces, as has been shown in schizophrenia. Importantly, however, this deficit seems to be corrected by later processing, as was indicated by behavioral responses. The early visuosensory processing of faces (P100 component) looks to be intact in schizotypy, although, investigation of the P100 response to face stimuli in individuals at various stages of psychosis-proneness would be profitable. To our knowledge, this is the first study to demonstrate that neurophysiological deficits in basic face processing are present in schizotypy. This work thereby adds to the mounting evidence for analogous neural processing anomalies at the healthy end of the psychosis continuum. The N170 deficits shown by high schizotypes in our study were present without the influence of confounds commonly associated with schizophrenia samples: such as repeated hospitalization, long-term antipsychotic therapy, social isolation, chronic neuropsychological profile, and, in many cases, lowered IQ. This confirms that N170 deficits reported previously in schizophrenia samples do not stem from these confounds. The findings further suggest that face processing deficits indexed by the N170 component may constitute neural dysfunction associated with vulnerability for schizophrenia (e.g., an endophenotype). This adds to the developing profile of individuals at a high risk for the disorder and may help facilitate their early detection. Finally, the results provide further evidence of underlying neurophysiological deficits that may contribute to the poor social interaction characteristic of schizophrenia.

## Conflict of Interest Statement

The authors declare that the research was conducted in the absence of any commercial or financial relationships that could be construed as a potential conflict of interest. The Associate Editor Dr. Caroline Gurvich declares that, despite having collaborated with author Dr. Susan L. Rossell, the review process was handled objectively and no conflict of interest exists.

## Footnotes

^1^The remaining three dimensions reflect positive and negative, but not cognitive, symptoms.

^2^The disorganized stimuli were included to provide a task and are not relevant to analyses.

^3^Tasks one and two had an imbalance of original stimuli due to the availability of images from the Ekman and Friesen series (91). The total number presented per condition was matched as closely as possible.

^4^Due to the difficulty of perceiving a Mooney face in the inverted position, 20 trials were considered a reasonable cut-off.
